# Testing the circulation of expanded flaps—prevention of necrosis of expanded flaps (a clinic study)

**DOI:** 10.3389/fped.2022.976150

**Published:** 2023-01-26

**Authors:** Tao Han, Haini Chen, Jianbin Chen, Jie Cui, Weimin Shen

**Affiliations:** Department of Burns and Plastic Surgery, Children's Hospital of Nanjing Medical University, Nanjing, China

**Keywords:** testing the circulation, expanded flap, flap necrosis, children, blood supply

## Abstract

**Background:**

Expanded flaps are commonly used in plastic surgery. Although expanded flaps are more resistant to hypoxia than unexpanded flaps, flap necrosis can sometimes occur, particularly with skin incisions of regular proportion. Distal skin necrosis of the expansion flap can be avoided by careful design; however, the utilization rate of the expansion flap decreases. Consequently, successfully avoiding distal skin flap necrosis remains a challenge. In this study, we designed a device for testing the circulation of the expanded flap that can decrease the risk of expanded flap necrosis, thus maximizing the use of an expanded flap.

**Methods:**

A total of 128 patients who underwent surgical repair between 2011 and 2019 and were retrospectively examined with the device for testing the circulation of the expanded flap were included in the study. The procedure included (1) making a device for testing the circulation, (2) implanting a skin expander, (3) injecting normal saline into the skin expander, (4) testing the circulation of the expanded flap, and (5) transferring the expanded flap to repair the defect.

**Results:**

One hundred forty-eight expanded flaps were implanted in 128 patients. The expanded flap that was transferred to repair the defect had no necrosis or infection. None of the expanded flaps with separated blood supply, which could be observed during operations, revealed complications. The survival rates of the expanded flap were increased by testing the circulation of the expanded flap. Expanded flaps designed by this method showed no swelling or paleness and no obvious temperature changes. In addition, the length-to-width ratio could be extended to 3:1.

**Conclusions:**

Our proposed method resulted in an effective surgical procedure for the repair of tissue defects. This approach could effectively change the direction of the blood vessel of the expanded skin flap and prevent necrosis of the expanded flap, thus representing a practical way to increase the use of expanded flaps and the flap survival rate, making the whole expanded flap transfer procedure more convenient.

## Introduction

Conventional skin reconstruction methods contain skin grafts and transfer of skin flaps. Skin grafts can produce an inconsistent skin change color, while the transfer of skin flaps can produce necrosis of transferred skin flaps. Filatov wrote the first paper on tubed pedicle formation in 1917. The method provided a technology for neovascularization and skin reconstruction. However, there was extensive scarring in the tubed region.

Tissue expansion is a technology of skin reconstruction. In 1984, Mander et al*.* (1) reported the expansion of adjacent tissue for scar resurfacing. Its advantage was repairing skin defects with neighboring skin, which was similar in color and texture and could avoid secondary damage to the donor site. Yet, in some cases, the utilization rate of the expanded skin flap is not high, along with flap necrosis. Namd found that flap necrosis easily occurs, and the width of the expander must be twofold larger than that of the defect. Necrosis of the skin flap was also reported in other papers (2, 3).

**Figure 1:**
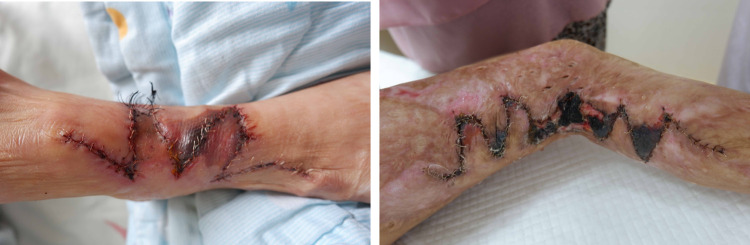
Necrosis of the flap tip.

We have noted that partial flap necrosis still occurs in our practice (Figure 1). Flap necrosis is extremely undesirable for patients, particularly on the face and limbs. Many surgeons would prefer to choose conventional graft skin rather than resolve these issues. It was also reported that surgical delay could prevent flap necrosis, which has been proven reliable for increasing the vascularity of a random flap (4). Thus, the main problems we have to solve are increasing the blood supply and maximizing the usage of the expanded flap.

Testing the circulation is an old technology of training skin blood flow for tube flap. In this study, we designed a device for testing the circulation of the expanded flap that can decrease the risk of expanded flap necrosis, thus maximizing the use of an expanded flap.

## Patients and methods

### Patients

A total of 128 patients who underwent surgical repair between 2011 and 2019 were retrospectively examined with a testing circulation device. There were 66 men and 62 women patients, with an average age of 38 months. There were 88 cases with scars, 22 cases associated with a congenital giant nevus, and 18 cases of body surface tumors (neurofibroma, hemangioma, fibromatosis, melanoma). One hundred forty-eight expanded flaps were implanted in 128 patients. Evaluation results were recorded.

### Device for testing the circulation

**Figure 2:**
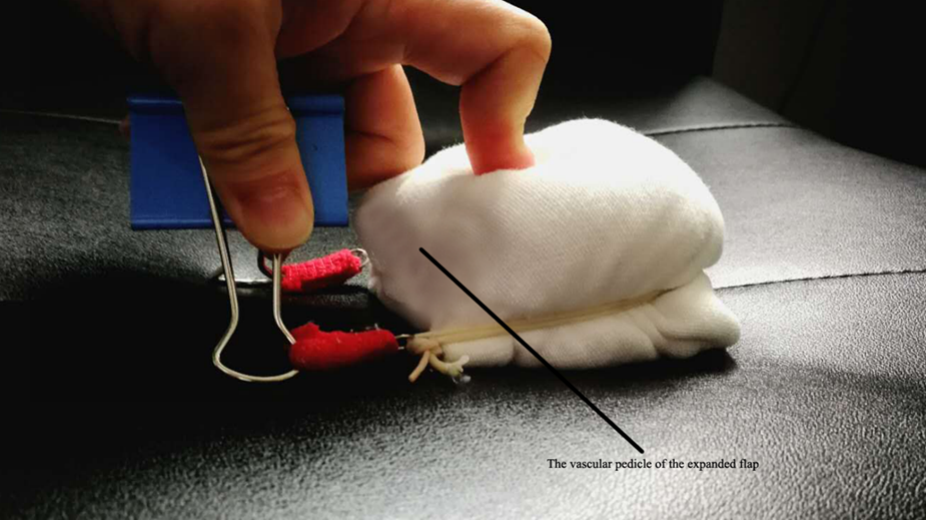
Device for testing the circulation of the expanded flap.

**Figure 3:**
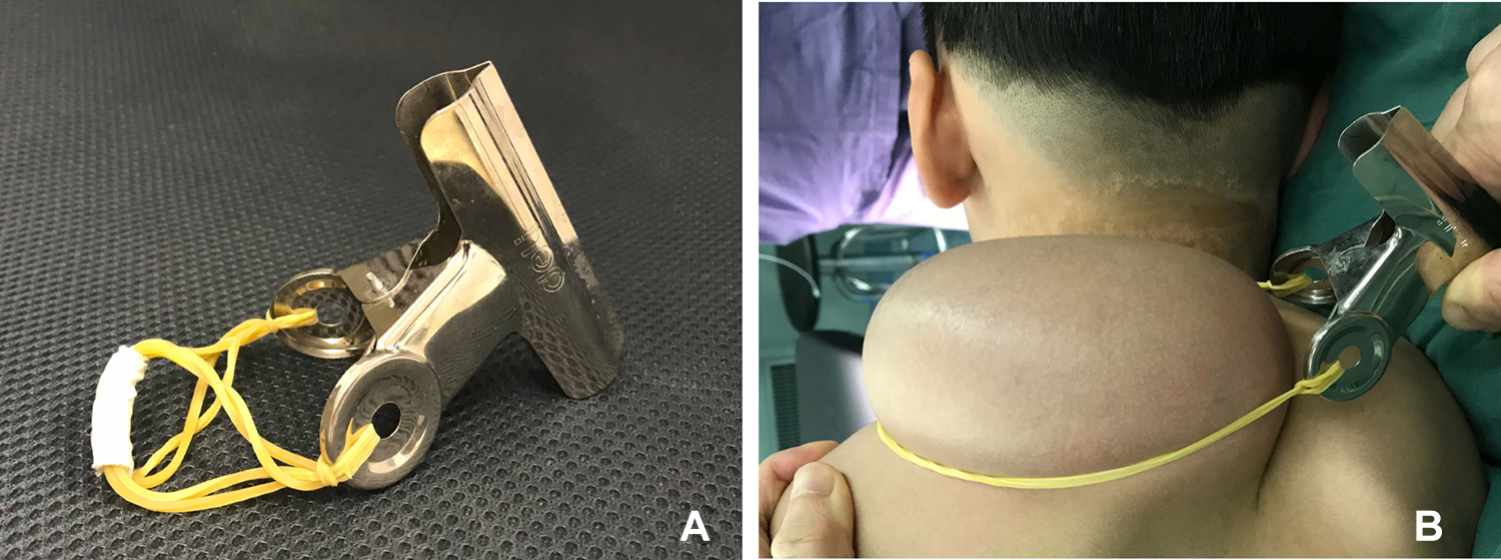
Testing the circulation of the expanded flap. (**A**) Device for testing the circulation. (**B**) Testing the circulation of the expanded flap.

The device for testing the circulation consisted of a tourniquet and a bridge of vascular pedicle pass. The tourniquet was made of a general rubber strip. The bridge was made of a plastic plate and covered the vascular pedicle. The vascular pedicle pass was made into the corresponding size according to the bridge's height (Figures 2, 3).

### Operation technique and testing the circulation of the expanded flap

All patients with implanted expanders underwent surgical repair followed by standard general anesthesia and received one dose of intravenous antibiotic coverage half an hour before the procedure.

### Expander implantation

The incision was carefully performed at the edge of the defect, with the expander package forming below the superficial fascia. The size of the expander ranged from 100 to 400 ml. The expander package was at least 1–2 cm larger than the expander in each dimension. The tissue expander was implanted into the prepared package, while the filling port was placed in a performed cavity with no potential pressure next to the expander site. In all cases, we only used remote internal filling ports and base tissue expander and made sure to smoothen the connection between filling ports and expander. After the insertion of the expander, the package was closed with three layers of sutures. The inner layer was sutured by a 4-0 absorbable stitch, while subcutaneous tissue was sutured by a 6-0 absorbable stitch. A 7-0 nylon stitch was used to suture the skin. We did not place any drainage in any expander package.

### Expander inflation

The expander inflation process started on the seventh day after surgery. Saline was injected every 3 days, and the final volume of injection was decided based on the size of the expander. The injecting volume each time was 5%–10% of the designed volume of the expander. The skin color was carefully observed, capillaries were refilled, and expander palpation was performed before and after injection.

### Testing the circulation of the expanded flap

**Figure 4:**
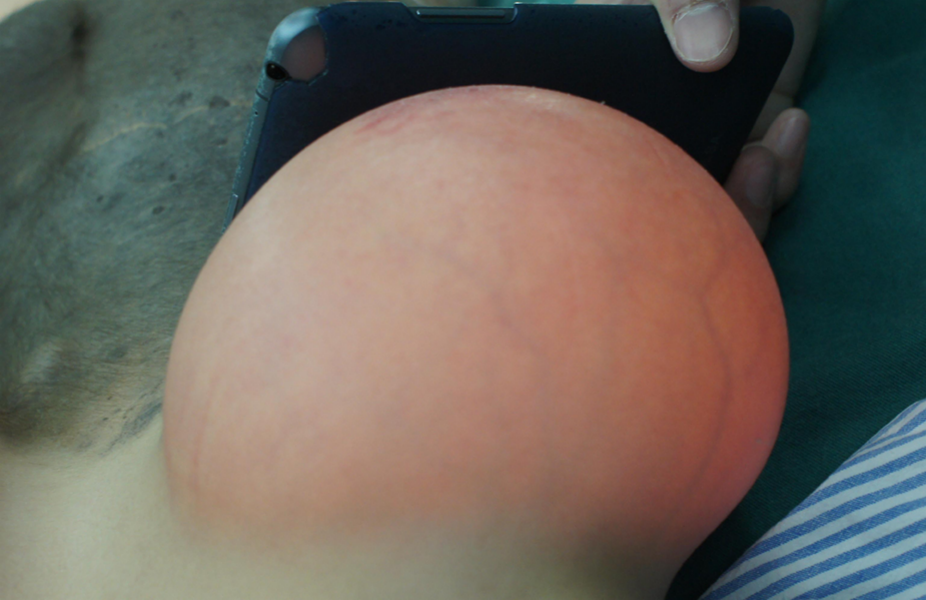
Expanded flap being ready for repair surgery.

The patients were ready to undergo testing of the circulation of the expanded flap 1 month after the expansion was completed. First, the rubber strip was clamped to block the blood supply for 10 min. If there was no color change of the expanded flap skin, the clamping time could gradually be extended, and the clamping could be performed 4–6 times daily. Finally, when the clamping time could last 8 h and the expanded flap's skin showed no swelling or paleness and no obvious temperature change, the promotion of blood circulation was considered successful, and the expanded flap was ready for repair surgery (Figure 4). To facilitate patients to finish circulation testing by themselves, online video tutorials were performed during postoperative follow-up.

### Design and rotation of the expanded flap

**Figure 5:**
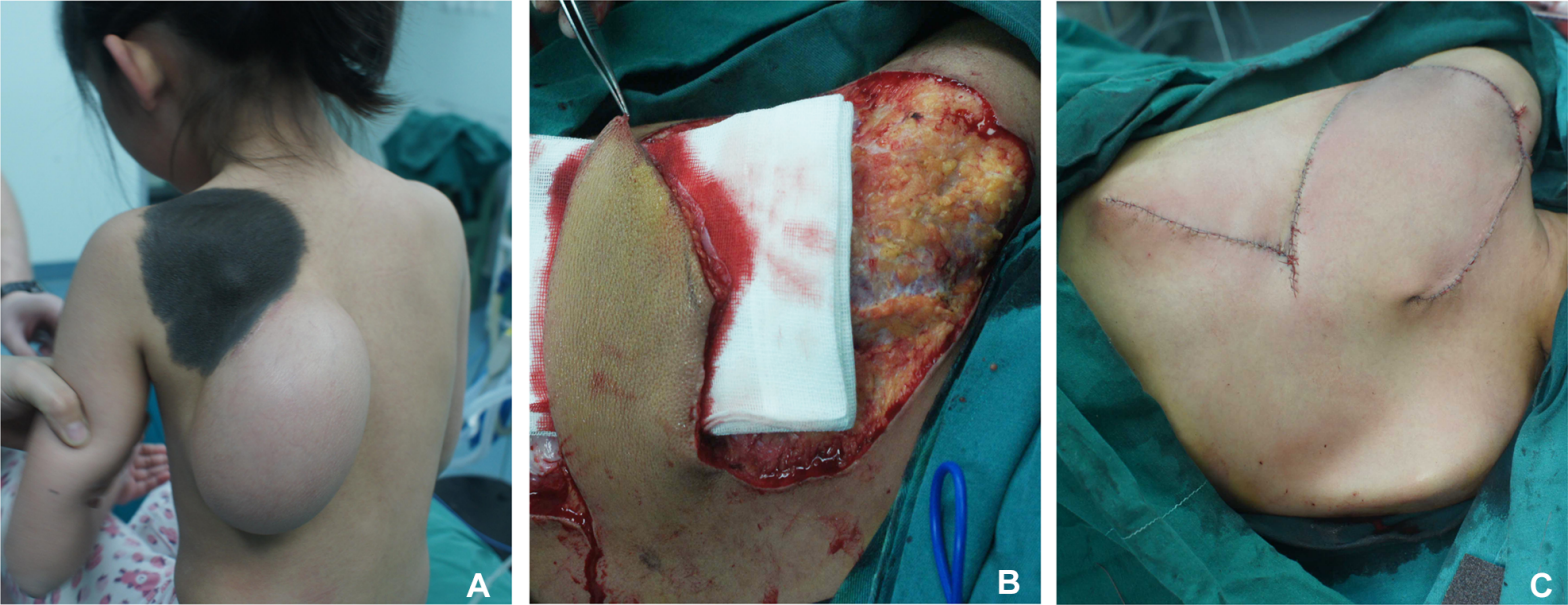
Skin incision was made along the designed line on the expanded flap, and length-to-width ratio of the flap was 1:3. (**A**) Preoperative appearance. (**B**) Length-to-width ratio of the flap was 3:1. (**C**) Immediate postoperative appearance.

According to the newly reformed circulation by our technique and defect shape, we designed the shape of the rotation flap. A skin incision was made along the designed line, and the length-to-width ratio was 3:1 (Figure 5).

## Results

One hundred forty-eight expanders were implanted in 128 patients. No hematoma, no expander exposure, and no infection were reported. All expended flaps were randomly rotated without any necrosis. There were two cases of distal skin cyanosis, which eventually improved and finally healed after repeated wound dressing changes. There was no obvious scarring in any of the cases after repair surgery.

### Case 1

**Figure 6:**
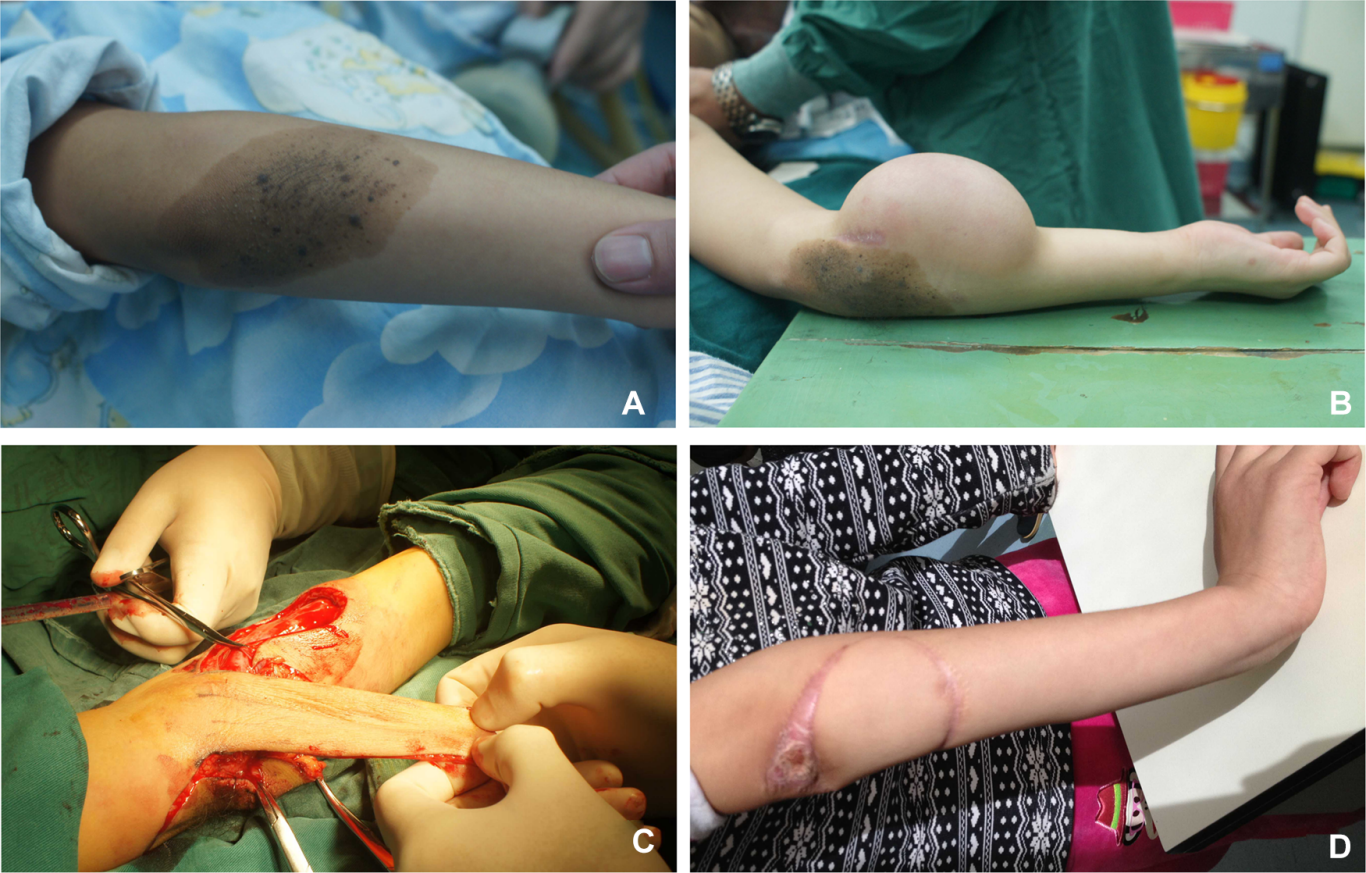
Case 1. (**A**) Upper limb moles. (**B**) Preoperative appearance of the expanded flap. (**C**) Flap resection during operation. (**D**) Postoperative appearance at follow-up.

The patient was a 5-year-old boy with a 15 × 30 cm congenital nevus on his left upper limb. He was implanted with one reniform-shaped tissue expander (200 ml) between the subcutaneous tissue and fascia below the nevus. Then, 20 ml of normal saline was injected into each expander every 3 days until it was inflated to 400 ml in volume over 2 months (Figure 6A). We started testing the circulation of the expended flap from 10 min to 1 h for the first 2 days and extended the duration to 2 h from the fourth day onward. The clamping was performed 4–6 times each day. Finally, when the clamping time was more than 8 h, we transferred the expanded flap to repair the defect after the nevus was removed, and the flap survived without necrosis (Figure 6B–D).

### Case 2

**Figure 7:**
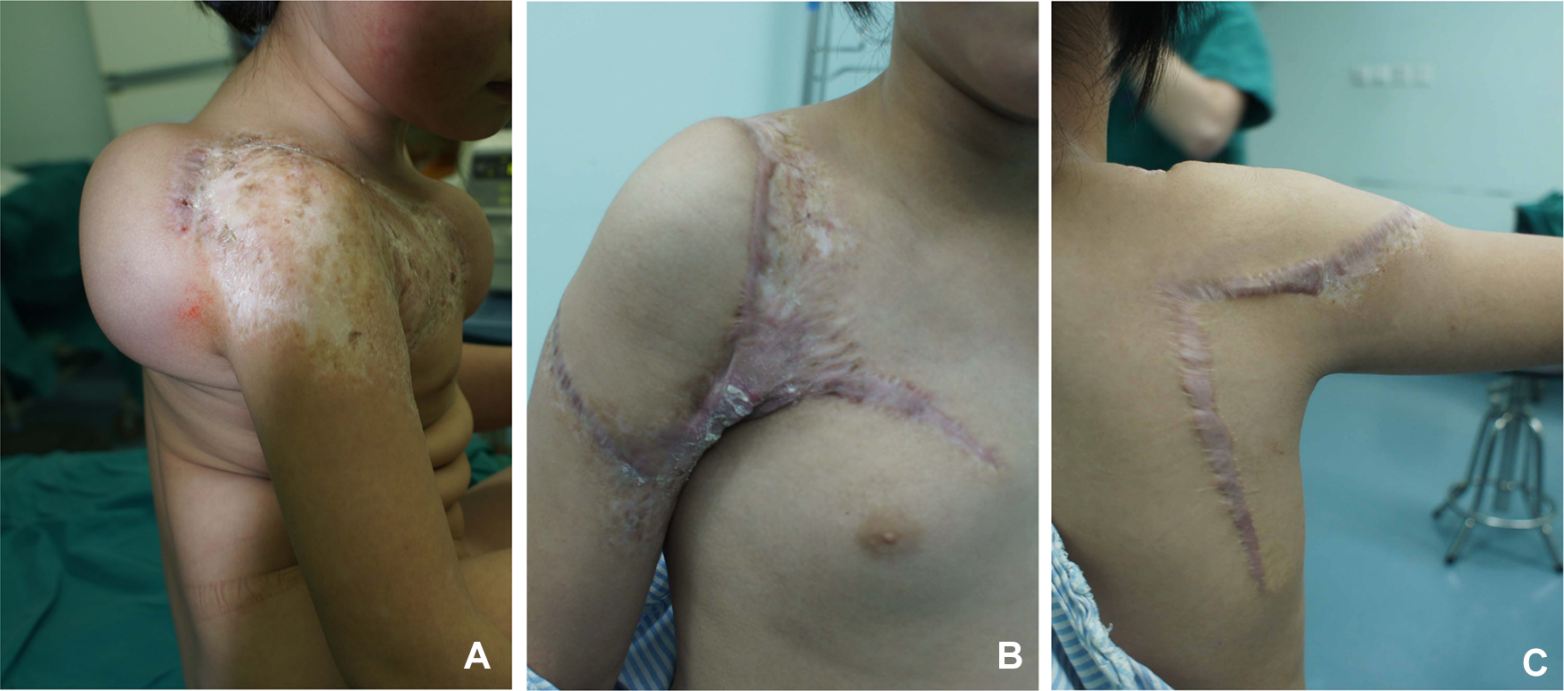
Case 2. (**A**) Preoperative appearance of the expanded flap. (**B**) Anterior view at postoperative follow-up. (**C**) Posterior view at postoperative follow-up.

A 6-year-old girl presented with a scar on the right shoulder she had since birth. A 400-ml reniform-shaped tissue expander was implanted between the subcutaneous tissue and the superficial muscular aponeurotic system in the shoulder. The expender was serially inflated with saline to 800 ml in volume over 3 months. We started testing the circulation of the expended flap following our protocol and rotated the flap to repair the defect after the vascular malformation was removed. The flap was not affected by necrosis (Figure 7).

### Case 3

**Figure 8:**
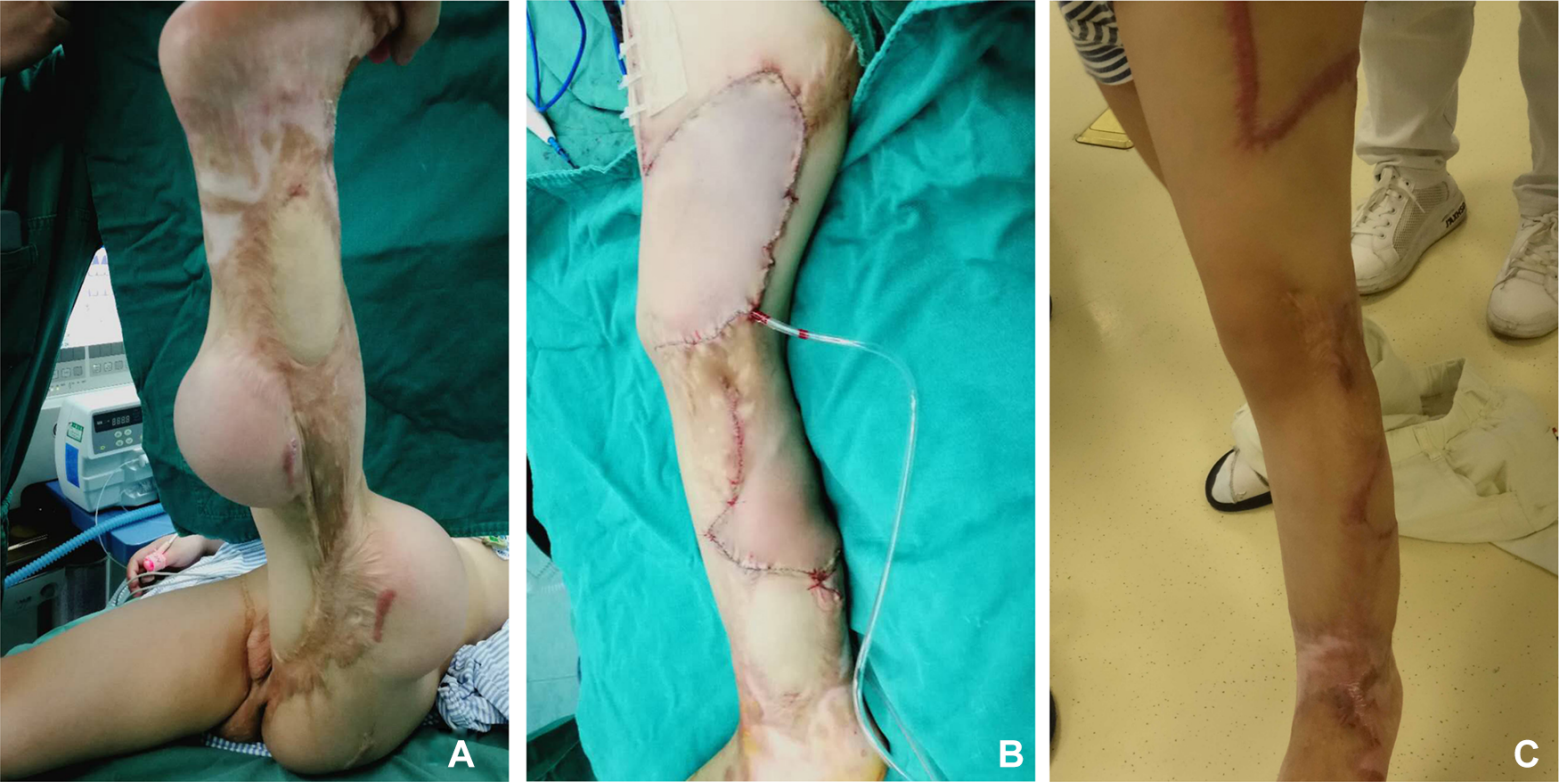
Case 3. (**A**) Preoperative appearance of the expanded flap. (**B**) Immediate postoperative appearance. (**C**) Postoperative appearance at follow-up.

A 3-year-old girl who presented with an 18 × 22 cm scar on her left lower limb was referred to our center. A reniform-shaped tissue expander (200 ml) was implanted between the subcutaneous tissue and fascia above the scar, while another expander was implanted below the scar. We inflated the expander to 400 ml over 3 months and performed testing of the circulation of the expanded flap following our protocol (Figure 8A). The flap was randomly rotated to repair the defect with a good outcome (Figure 8B,C).

## Discussion

Skin soft-tissue expansion first began in 1976, and over a period of 40 years, it has become widely used in various fields of plastic surgery. It has become one of the basic plastic technologies highly used for tissue repair, skin grafting, and skin flap transfer (5).

Skin soft-tissue expansion is a method that involves implanting the expander under normal skin and soft tissue and increasing the pressure on local skin by inflating the expender in volume, thus creating the new “extra” skin and transferring it to repair the defect. Skin soft-tissue expansion is widely used in repairing skin or soft tissue defects induced by various causes, such as incision of congenital giant nevus, scars, tumors, and skin defects after trauma. Following the wide application of expanders, numerous studies have reported an increasing number of expanded skin flap uses (6–8). However, the use of a flap has also been associated with some complications such as necrosis, infection, and hematoma. Hu et al. (9) suggested that the necrosis of the expanded flap was related to its length-to-width ratio and the presence or absence of well-known arteries. Moreover, Chen and colleagues (10) argued that changing the blood flow of the expanded flap could prevent the necrosis of the flap. Therefore, increasing the blood supply, maximizing the usage of the expanded flap, and preventing necrosis after flap rotation surgery are challenging issues that our proposed method can successfully overcome.

By testing the circulation of the expanded flap, we can change the vascular distribution of the skin and achieve blood vessel axial development. Applying this kind of skin flap can exceed the normal ratio (11, 12) and evaluate vascular redistribution (13). Yet, Hainan et al. (14) reported on using prefabricated expanded skin flaps, revealing a significantly lower rate of necrosis after flap rotation surgery. This provided a new approach for preventing the expended flap from necrosis; however, further research on axial pattern skin flaps is still urgently needed. With vascular transformation emerging in the tube flap through surgery or blood supply training, we can get similar outcomes to the tube flap if we perform blood supply training on the expended skin flap using the principle of the tube flap. Our research showed that vascular transformation occurs in an expended flap after long-term blood supply training with our testing circulation device. The direction of a blood vessel on the expended flap can change as the designed position of the vascular pedicle pass. We could design flap transfer in the way of an axial pattern skin flap, which in turn could increase the use of expanded flaps and the flap survival rate, making flap transfer surgery more convenient. In our experience, if there is no necrosis as the elasticity of the tourniquet increases to the maximum for 6 to 8 h, the expended flap will survive without necrosis after flap transfer surgery. Future experimental research is needed to further verify whether the elasticity of the tourniquet may block the blood supply of the expended flap.

## Data Availability

The original contributions presented in the study are included in the article/Supplementary Material; further inquiries can be directed to the corresponding author/s.
